# The Spatiotemporal Dynamics of Cerebral Autoregulation in Functional Magnetic Resonance Imaging

**DOI:** 10.3389/fnins.2022.795683

**Published:** 2022-07-08

**Authors:** Joseph R. Whittaker, Jessica J. Steventon, Marcello Venzi, Kevin Murphy

**Affiliations:** ^1^Cardiff University Brain Research Imaging Centre (CUBRIC), School of Physics and Astronomy, Cardiff University, Cardiff, United Kingdom; ^2^Max Planck Institute for Human Cognitive and Brain Sciences, Leipzig, Germany; ^3^Cardiff University Brain Research Imaging Centre (CUBRIC), School of Medicine, Cardiff University, Cardiff, United Kingdom

**Keywords:** cerebral autoregulation, fMRI, resting-state, thigh-cuff manoeuvre, blood pressure, cerebral physiology, BOLD

## Abstract

The thigh-cuff release (TCR) maneuver is a physiological challenge that is widely used to assess dynamic cerebral autoregulation (dCA). It is often applied in conjunction with Transcranial Doppler ultrasound (TCD), which provides temporal information of the global flow response in the brain. This established method can only yield very limited insights into the regional variability of dCA, whereas functional MRI (fMRI) has the ability to reveal the spatial distribution of flow responses in the brain with high spatial resolution. The aim of this study was to use whole-brain blood-oxygenation-level-dependent (BOLD) fMRI to characterize the spatiotemporal dynamics of the flow response to the TCR challenge, and thus pave the way toward mapping dCA in the brain. We used a data driven approach to derive a novel basis set that was then used to provide a voxel-wise estimate of the TCR associated haemodynamic response function (HRF_*TCR*_). We found that the HRF_*TCR*_ evolves with a specific spatiotemporal pattern, with gray and white matter showing an asynchronous response, which likely reflects the anatomical structure of cerebral blood supply. Thus, we propose that TCR challenge fMRI is a promising method for mapping spatial variability in dCA, which will likely prove to be clinically advantageous.

## Introduction

Cerebral blood flow (CBF) is arguably the most critically important physiological parameter with respect to neurological heath. The human brain has a very high metabolic rate, but a limited capacity for substrate storage, so continual delivery of fresh blood is a prerequisite for healthy functioning (Willie et al., 2014). Cerebrovascular control of blood flow occurs in many contexts, but the term cerebral autoregulation (CA) is usually reserved to refer to the self-evident process by which the cerebrovascular system reacts to fluctuations in systemic blood pressure to keep CBF relatively constant (van Beek et al., 2008; Willie et al., 2014). A distinction between static and dynamic modes of autoregulation is often made in the literature [sCA and dynamic cerebral autoregulation (dCA) respectively], which primarily reflects experimental conditions and the time-scale over which blood pressure changes are observed, rather than any formal suggestion of fundamentally different processes (Brassard et al., 2021). The term dynamic CA (dCA) was introduced following the development of Transcranial Doppler (TCD) ultrasound. This new technology allowed changes in cerebral blood flow velocity (CBFV) to be measured at high temporal resolution, and thus the CA response to the most transient of mean arterial blood pressure (MAP) fluctuations to be characterized (Aaslid et al., 1989). Short time scale fluctuations in MAP (on the order of seconds) can be experimentally induced with a variety of physiological challenges, including various types of orthostatic maneuver, tilt-table tests, and thigh-cuff-release (TCR) challenges (van Beek et al., 2008). Additionally, MAP also exhibits spontaneous fluctuations across a broad band of frequencies (Parati et al., 1995), including high frequency changes on a beat-to-beat basis. Thus, using TCD to record CBFV concurrently with spontaneous or induced MAP fluctuations has permitted the temporal dynamics of dCA to be well characterized. A host of analysis techniques have also been developed, all attempting to extract the physiologically relevant indices of dCA from the covarying MAP and CBFV time series (Panerai, 2009). This experimental paradigm has proved to be extremely clinically powerful, and there are many reports of altered dCA in a variety of patient groups (White and Markus, 1997; Panerai et al., 2004; Navarro et al., 2007; Aries et al., 2010).

However, the fundamental limitation of TCD is that it only supplies global flow information, albeit with excellent temporal resolution. Whilst undoubtedly powerful, particularly when studying a relatively systemic process such as dCA, we know that regional CBF is highly heterogeneous in humans (Henriksen et al., 2018). This intra-individual variability, which is fundamental to active neuronal functioning (Yuan et al., 2016) and forms the basis of functional magnetic resonance imaging (fMRI), is also relevant in the manifestation of cerebrovascular diseases such as dementia (Morgan et al., 2021). Thus, it is not unreasonable to conclude that the spatial pattern of activity should not be discounted when characterizing dCA, especially in the context of disease. To this end, fMRI techniques appear to offer the most promise, as they allow flow related information to be measured with high spatial resolution and whole-brain coverage. Of the set of physiological challenges that have been used to assess dCA, those based on gross whole-body physical movements (e.g., squat-stand maneuvers or tilt-tables) are clearly incompatible with the MRI. We have successfully experimented with using lower body negative pressure (LBNP) to measure sCA in a previous study (Whittaker et al., 2019a), but the technically demanding experimental requirements present a considerable challenge for widespread clinical research. Furthermore, the significant subject motion associated with LBNP precludes it as an effective means for measuring dCA. We have also shown that endogenous fluctuations in MAP are correlated with fMRI signals (Whittaker et al., 2019b), which we have suggested is related to autoregulatory processes. However, these endogenous MAP fluctuations are naturally lower in magnitude compared with induced ones, which not only has implications for SNR, but may also challenge the ecological validity (i.e., generalizability to real-life scenarios) of using them to characterize dCA (Simpson and Claassen, 2018).

However, the literature does include numerous studies, which demonstrate the feasibility of employing TCR challenges to measure dCA in the MR environment (Saeed et al., 2011; Horsfield et al., 2013; Panerai et al., 2016). These important earlier studies prove that fMRI is sensitive to autoregulatory responses, however they almost invariably characterize dCA using only global average time series, and so do not fully exploit the main advantage of fMRI over TCD, i.e., the ability to spatially resolve flow responses in the brain. Although (Horsfield et al., 2013) did attempt to look at regional differences, they used a model fitting procedure developed from TCD data, which accounts for an initial signal drop and subsequent recovery. Thus, this TCD originated method is likely to be suboptimal for use with the fMRI signal, and may not be capable of revealing the full spatiotemporal dynamics of the fMRI dCA response. Advances in technology such as partially parallel imaging (Pruessmann et al., 1999; Griswold et al., 2002) and simultaneous multi-slice (SMS) (Feinberg et al., 2010; Moeller et al., 2010) now allow fMRI to achieve reasonably good temporal resolutions with whole-brain coverage. Thus, the purpose of this study is to explore the use of simultaneous blood-oxygenation-level-dependent (BOLD) fMRI and TCR to characterize the spatiotemporal dynamics of dCA in the healthy human brain with whole-brain coverage.

## Materials and Methods

### Experimental Protocol

Ten healthy male volunteers (aged between 20 and 41 years) were recruited for a single session. All volunteers gave written informed consent, and the Cardiff University School of Psychology Ethics Committee approved the study in accordance with the guidelines stated in the Cardiff University Research Framework (Version 4.0, 2010). For each subject fMRI scans were performed under two conditions. (1) TCR conditions coinciding with a TCR maneuver as outlined below (*tcr-fMRI*). (2) Rest conditions; during standard resting-state conditions (*rs-fMRI*).

#### Imaging Protocol

A Siemens 3T MAGNETOM Prisma scanner equipped with a 32-channel receive head-coil was used to acquire data. The Centre for Magnetic Resonance Research (CMRR) multiband sequence was used to acquire SMS gradient-echo echo-planar imaging (EPI) BOLD fMRI data with the following parameters; TR = 1 s, TE = 30 ms, flip-angle = 58°, 2 mm isotropic resolution, 60 slices, multiband factor = 4, GRAPPA = 2. For 9 of the 10 subjects, 5 repeated *tcr-fMRI* scans were acquired, whereas for the first subject 8 repeats were acquired. For the first 2 subjects 60 volumes were acquired, whereas for the remaining 8 subjects 90 volumes were acquired. This adjustment was made after the beginning of the study to account for the possibility that a long scan time may be needed to measure the full dynamics of the response. In addition to *tcr-fMRI* scans, for each subject a *rs-fMRI* scan was also acquired using the same parameters but with 600 volumes (i.e., 10 min long). The rest scans were acquired immediately after the last *tcr-fMRI* scan. Finally, a standard T_1_-weighted MPRAGE scan was acquired for each subject for registration purposes (FOV = 220 mm, TR = 2.2 s, TE = 3 ms, TI = 1.05 s, α = 8°, 224 continual sagittal slices, 0.7 mm isotropic resolution, 6 m 53 s acquisition time). Concurrent physiological traces were recorded for all runs, including photoplethysmography (PPG), capnography for measuring expired partial pressure of end-tidal carbon dioxide (P_*ET*_CO_2_), and beat-to-beat blood pressure *via* the MR compatible Caretaker system (Biopac, United Kingdom).

#### Thigh-Cuff Release Challenge Protocol

**FIGURE 1:**
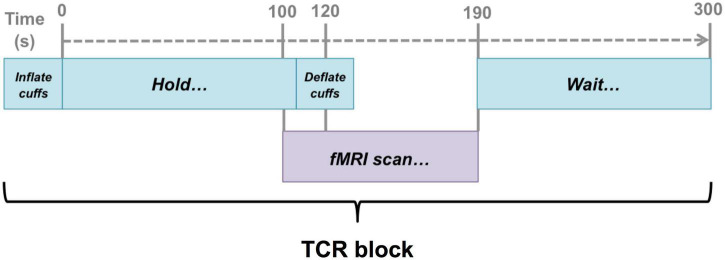
Schematic illustration of the timing of the fMRI scans with respect to the TCR challenge. Scans were timed such that they captured a 20 s baseline before the TCR onset, and then allowed 70 s for the TCR response to evolve (40 s in the case of 2 subjects).

For each subject repeated *tcr-fMRI* scans were acquired according to the protocol depicted in the schematic in Figure 1, with the Vicorder system (Skidmore Medical, United Kingdom) used to deliver the TCR challenge. For each subject, once positioned on the scanner bed, pneumatic cuffs were placed around each thigh at a level so that the upper edge of the cuff was approximately at the level of the most inferior portion of the gluteus maximus muscle (i.e., cuffs were placed on the upper thighs). Care was taken to ensure that cuffs were firmly secured and tightly fixed without discomfort for the participant. An additional standard blood pressure cuff was placed around the arm to take blood pressure measurements using an MRI compatible monitoring system (Veris MR, MEDRAD, PA, United States). Once the subject was positioned and ready to be moved into the bore of the scanner, they were given a few minutes to relax and adjust to the MR environment. Once the subject was ready to proceed with the experiment blood pressure readings were obtained to provide a reference for determining the cuff inflation pressure for the TCR challenge. Two readings were taken to ensure that the subject had reached a steady-state and had fully acclimatized to being in the supine position, and the second reading was then used as the reference measure.

For each subject and for each TCR maneuver the thigh cuffs were inflated to 40 mm Hg above the reference systolic blood pressure. Before the experiment began, one full run through of the TCR maneuver was completed to ensure that the participant could tolerate the challenge. All subjects tolerated the TCR-challenge and none experienced any significant pain. Some mild discomfort was reported, but never sufficiently severe such that any subject chose not to continue the experiment.

Once in the scanner fMRI data were acquired to capture the deflation period of the maneuver. The same basic procedure was repeated according to the schematic as shown in Figure 1. First the cuffs were inflated to the target level of 40 mm Hg above the reference systolic blood pressure. Once the target level was reached, the cuffs were held inflated at that pressure for 120 s before being rapidly deflated. The fMRI scan was timed such that data collection began 20 s before the deflation occurred. Once data collection was complete a period of 110 s of rest was given before the whole 300 s sequence was repeated.

### Analysis

Main analysis code is available *via* this GitHub repository: https://github.com/jrwhittaker/tcr_fmri_analysis_scripts.

#### Pre-processing

Both *tcr-fMRI* and *rs-fMRI* data were preprocessed and registered to MNI space with a pipeline created using AFNI (Cox, 1996), FSL (Jenkinson et al., 2012), and ANTS (Avants et al., 2009). Briefly, it consisted of the following steps. (1) De-spiking and motion correction; (2) RETROICOR to remove cardiac and respiratory cycle related variance; (3) Non-linear registration to MNI space. (4) Regression to remove noise variance using a CSF nuisance regressor (see Supplementary Figure 1 for time series) and 6 motion parameters, which was combined with 0.01–0.1 Hz bandpass filtering (*rs-fMRI* only) in a single step using AFNI’s *3dTproject* function; and (5) Averaging together of *tcr-fMRI* repeats into a single scan to remove non TCR-challenge evoked signal fluctuations. Concurrent physiological recordings were processed to yield heart rate, MAP, and P_*ET*_CO_2_ time series as percentage signal change from the baseline period prior to the TCR onset.

#### Novel Custom Basis Set

As is customary with the response to a brief neural stimulus, we assume that the BOLD signal response to the TCR-challenge (ΔBOLD_*TCR*_) is the output of a linear time-invariant (LTI) system, meaning it is both time invariant and of finite duration. Thus, in treating the TCR-challenge evoked transient MAP response as an impulse input to the system, we can estimate the impulse haemodynamic response function (HRF) to the TCR-challenge (HRF_*TCR*_). Although fMRI studies typically use a canonical HRF that is assumed to be homogenous across the brain, this approach precludes the observation of regional differences in temporal dynamics. As our aim is to characterize the spatiotemporal dynamics of the TCR-challenge response we need a method that allows us to fit ΔBOLD_*TCR*_ at a voxel-wise level. However, ΔBOLD_*TCR*_ responses may be quite noisy and contain considerable within and between subject variability. Furthermore, we have no *a priori* assumptions regarding the form the response should take, and the canonical HRF is unlikely to be appropriate given that the mechanisms underlying the TCR-response will likely be very different.

**FIGURE 2:**
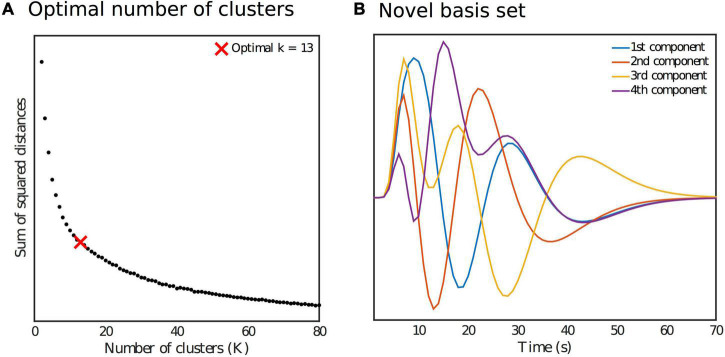
**(A)** Identification of the optimal number of clusters k for the k-means reduction step as part of the generation of the novel basis set. **(B)** Orthonormal novel basis set.

We therefore used a data-driven approach to derive a novel basis set, which generalizes sufficiently well to account for within and between subject variability, whilst minimizing the risk of over-fitting. For each subject the Harvard-Oxford cortical atlas was used to extract 48 cortical ROI time series from the mean *tcr-fMRI* dataset, resulting in a total of 480 time series. We then fit a smooth curve to each time series from a series of gamma density functions as described in (Prokopiou et al., 2019), as given by


$$g\,\left(t,\tau \right)=\left\{ \begin{array}{c} \begin{array}{cc} e^{-t\,\sqrt{\sigma \,\tau }}\,{\left(\frac{e\,t}{\tau }\right)}^{\sqrt{\frac{\tau }{\sigma }}} & t\geq 0 \end{array}\\ \begin{array}{cc} 0\; & t<0 \end{array} \end{array}\right.$$

where τ and σ determine the peak’s location and width, respectively. A set of 8 such functions was used as regressors in order to fit smooth ΔBOLD_*TCR*_ responses for every ROI time series within a GLM. The parameter values used to generate the regressors were varied so as to cover the range of temporal dynamics in the expected ΔBOLD_*TCR*_ response. This resulted in a set of 480 smooth response curves (10 subjects times 48 cortical ROIs) spanning the range of possible regional and subject specific response shapes within the sample. Of these, the top 50% (240) best fitting curves (according to *R*^2^) were selected, and then a further dimensionality reduction step was performed on this set of smooth curves by grouping them into several clusters using the k-means clustering algorithm. The elbow method was used to determine the optimal number of clusters (13) from the sum of square distances as shown in Figure 2A, with the elbow of the curve defined as the data point with the largest Euclidean distance from the line connecting the first and last points. Finally, we applied singular value decomposition (SVD) to the set of cluster averaged response curves. The first four singular vectors explained 91% of the variance in the data, and so were taken as our orthonormal novel basis set (Figure 2B).

#### Voxel-Wise Fit

The spatiotemporal dynamics of the TCR response were characterized within a general linear model framework using a model that included the novel basis set, a linear drift term and an intercept. A second model was fitted that included P_*ET*_CO_2_ traces to account for any effect of blood CO_2_ concentration on the HRF_*TCR*_. The fitted response was then averaged across subjects to obtain an estimate of the group level spatiotemporal dynamics. To explore temporal dynamics within different tissue types, gray (GM) and white matter (WM) masks were estimated for each subject from partial volume estimates (PVE) derived from the T_1_-weighted image, based on a criterion of PVE >95% for each tissue type. To explore whether the temporal dynamics in WM would show a differential effect based on depth (i.e., superficial vs. deep WM), a series of erosion and subtraction calculations were then applied to the WM masks to generate 4 sub-masks that reflect proximity to the cortical ribbon. The most superficial layer mask was created by subtracting a WM mask that had one erosion performed from the total (un-eroded) WM mask. Subsequent layers then follow this same pattern of subtracting a more eroded mask from a less eroded mask. These sub-masks are characterized as *superficial* WM, *mid-superficial* WM, *mid-deep* WM and *deep* WM. Group averaged fitted responses were then extracted from these masks. Finally, to identify any coherent spatial structure in the HRF_*TCR*_ we applied k-means clustering to the group average voxel-wise fitted responses after spatial smoothing (Gaussian kernel, FWHM = 5 mm). The cluster number *k* was varied between the minimum of 2 and a maximum of 6. Visual inspection showed that choosing *k* > 6 yielded diminishing returns in terms of highlighting structured spatial variance.

#### Comparison of Thigh-Cuff Release-Functional Magnetic Resonance Imaging and Resting-State-Functional Magnetic Resonance Imaging

We would like to be able to examine the temporal dynamics present in the *tcr-fMRI* data in a more quantitative way. However, we want our approach to remain relatively unconstrained, without specifying features of HRF_*TCR*_. To this end we have chosen to look at the lag-adjusted correlation with global signal (GS), as it should reveal the basic spatial pattern of the relative temporal structure of the HRF_*TCR*_. The uninformed nature of this approach has made it the preferred approach for extracting global spatiotemporal information from resting-state fMRI (Liu et al., 2017; Tong et al., 2019). This also allows us to make a simple comparison between the manifestation of spatiotemporal variance during the TCR and rest conditions, and thus elucidate the degree to which dCA associated processes manifest themselves in resting fMRI fluctuations. For each dataset the GS was defined as the average time series within a whole brain mask. Truncated subsets of the GS were time shifted it in TR increments (i.e., 1 s), from −10 s to + 10 s, and then correlated with truncated fMRI time series at every voxel to create a cross-correlation function. This cross-correlation function was then fit to a series of Legendre polynomial functions (until *R*^2^ > 0.95 or up to a maximum order of 10) to obtain a smooth curve, from which the maximum correlation could be robustly estimated.

Thus, for both *tcr-fMRI* and *rs-fMRI* datasets we derived the maximum lag-adjusted correlation with the GS and the index of the time lag. For both metrics the spatial correlation between *tcr-fMRI* and *rs-fMRI* data was then calculated, and a *t*-test was performed following Fisher transformation of the correlation values. We also considered how similar the overall pattern of functional connectivity is in the *rs-fMRI* data compared with *tcr-fMRI*. The average signal in the 48 cortical ROIs that comprise the Harvard-Oxford atlas was extracted for both conditions, and then the group average correlation matrix was calculated. The correlation matrices can be qualitatively compared for rest and TCR conditions, but to quantitatively assess their similarity we calculated the Spearman’s rank correlation coefficient between corresponding connectivity values, i.e., correlating the values in the upper triangle of the correlation matrices. The use of Spearman’s Rank correlation does not assume a linear relationship to assess similarity between conditions, and any similarity could be due to dCA associated processes manifesting in resting-state fluctuations.

## Results

### Thigh-Cuff Release Response

**FIGURE 3:**
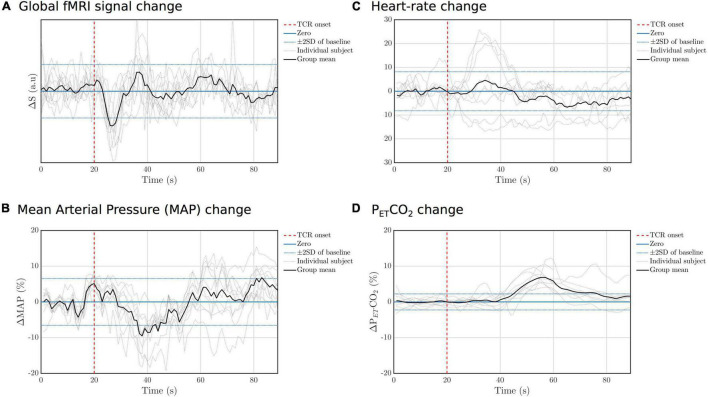
Individual subject and group mean TCR evoked responses for **(A)** GS, **(B)** MAP, **(C)** HR, and **(D)** P_*ET*_CO_2_. For each variable the standard deviation (SD) of the fluctuations during the baseline peroid (concatenated across subjects) was calculated and then lines added to the graph to indicate ± 2 standard deviations.

Temporal changes in the fMRI GS and other physiological parameters are shown in Figure 3, where a marked drop in the signal following the onset of the TCR can be seen. The MAP also shows a drop in amplitude following the TCR, although more slowly evolving compared with the fMRI GS. HR changes were highly variable across subjects, with no consistent response across subjects observed, whereas PETCO2 rose significantly in all subjects approximately 20 s after the TCR.

### Spatiotemporal Dynamics

**FIGURE 4:**
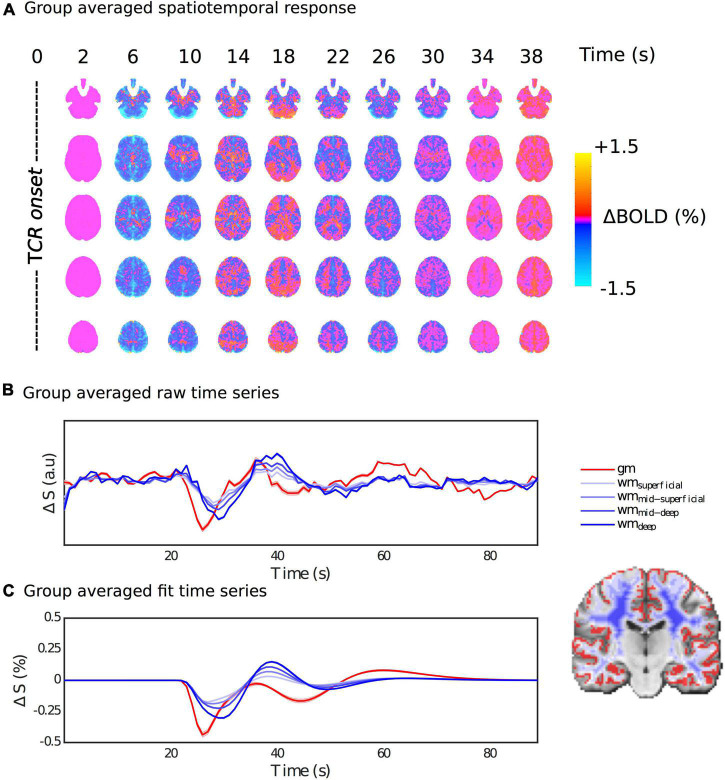
Spatiotemporal dynamics of the HRF_*TCR*_. **(A)** Percentage BOLD signal changes at multiple time points post TCR onset are shown in order to visualize the spatiotemporal variability of the HRF_*TCR*_. **(B,C)** Show raw and fitted responses, respectively, different tissue masks.

Figure 4 shows the group average fitted response at a series of time steps after the TCR challenge onset. Qualitatively one can see a clear spatiotemporal pattern, with a strong GM/WM contrast clearly manifesting at multiple time points within the full temporal evolution of the TCR response. In the Supplementary Material we include an additional Supplementary Figure 2, which shows that including P_*ET*_CO_2_ in the model makes very little difference to the overall spatiotemporal pattern, despite the TCR locked rise in CO2 we observe. This also suggests that the novel basis set is not significantly over-fitting the data. In the Supplementary Material we also include a Supplementary Figure 3 showing the fitted response at a series of time steps after the TCR challenge onset for the first 5 subjects. From this figure it can be observed that the same spatiotemporal pattern is present on an individual subject basis, and that it evolves in a similar manner for all subjects. However, it is also clear that there are differences between subjects. For example, subject 2 shows an initial widespread negative signal change across brain, in common with the other subjects, but it stays negative for longer.

**FIGURE 5:**
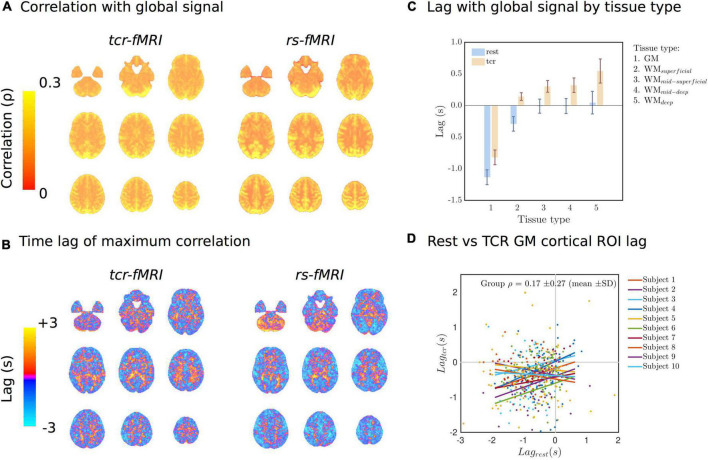
**(A)** Lag-adjusted correlation with the GS images for both conditions. **(B)** Lag time of the maximum correlation with the GS images. **(C)** Average lag time of the maximum correlation with the GS by tissue type. **(D)** Individual subject scatter plots of the Harvard-Oxford cortical ROI averaged lag times in Rest vs. TCR condition. Individual subject regression lines are included.

Figures 4B,C show the average raw time series and fitted response within GM and different subsets of WM, going from the most superficial to the deepest layers. In general, the WM response lags behind the GM one, but it also evolves as it propagates from superficial to deep layers. The magnitude of the WM response increases as it propagates from the superficial to the deep layers, and the lag with respect to GM appears to increase slightly too. This is also supported by Figure 5C, which shows the lag with respect to the global signal in the *tcr-fMRI* data also demonstrates this depth dependence in the temporal dynamics of the WM, and to a far greater degree than in the *rs-fMRI* data.

**FIGURE 6:**
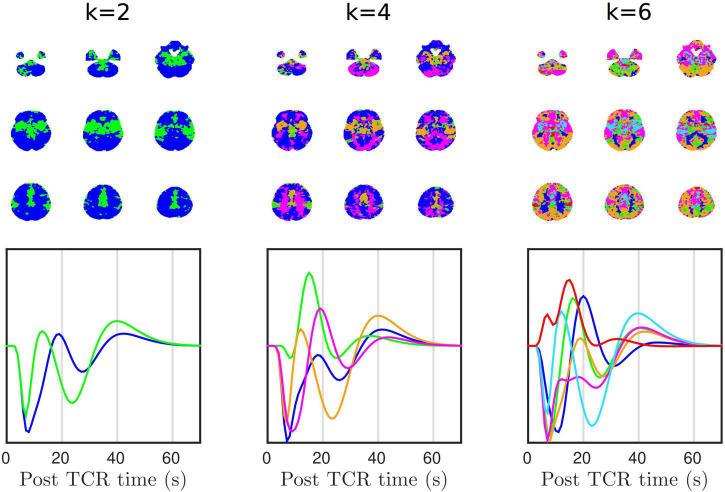
K-means clustering shows that there is structured spatial heterogeneity in HRF_*TCR*_.

In addition to the clear GM/WM disparity, more complex spatial patterns also emerge. Figure 6 shows that the HRF_*TCR*_ is organized into large-scale spatial patterns, which can be derived using k-means clustering. Simply selecting *k* = 2 clusters, we can see that the response can be separated into two distinct spatial patterns with similarly shaped, but unsynchronized, temporal dynamics. The two clusters do not appear to be delineated along GM/WM boundaries, but rather one cluster is more located in the middle of brain along the anterior-posterior axis, and so could reflect the vascular territory of the middle cerebral arteries, or alternatively the density of vascularization. At *k* = 4 clusters we start to see clearer GM/WM contrast, and at *k* = 6 cluster we start to see both GM/WM contrast as well as signs of cortical clustering, again perhaps partly representing specific gross vascular territories, but potentially more complex vascular spatial structure. It is also clear that individual clusters are reasonably symmetrical across the two hemispheres.

Additionally, we also performed a principle component analysis (PCA) of the group averaged *tcr-fMRI* data in order to identify the spatial patterns of the temporal components that account for most variance (Supplementary Figure 1). In contrast with *k* = 2 simple clustering, the spatial pattern of the 1st component predominantly reflects GM/WM contrast. However, the 2nd, 3rd, and 4th components show similar spatial patterns to the k-means clusters, with the 2nd component in particular showing high similarity with the *k* = 2 cluster separation. The 2nd component appears to reflect gross vascular territories, which reinforces our belief that they are a factor partially driving the k-means clustering.

### Comparison With Rest

Figure 5A shows the group average of lag-adjusted correlation with the GS for both *tcr-fMRI* and *rs-fMRI* datasets, and Figure 5B shows the group average of the lag time. For both conditions (TCR and rest) the spatial distributions of the lag-adjuster correlations with the GS show strong GM contrast, and there is a high degree of similarity between conditions with mean spatial correlation of ρ = 0.49 ± 0.08(*mean*±*SD*,*p* = 3.43×10^−8^), which means approximately 24% shared variance on average. The lag times show some similarity between the two conditions, particularly with regard to overall GM/WM contrast, but there appears to be more structured variability across the cortex in the rest condition, and the mean of spatial correlations ρ = 0.02 ± 0.02(*mean*±*SD*,*p* = 0.0012) is very low, albeit still significantly non-zero. Figure 5C shows the average lag by tissue type, and for both conditions there is a clear difference between GM and WM. For the TCR condition this lag also appears to be get progressively longer as a function of a WM depth relative to the cortical surface. Figure 5D is a scatter plot showing the relationship of Harvard-Oxford cortical ROI average lag times between the TCR and rest conditions for different subjects. It is clear from these plots and the individual subject lines of best fit that there is no strong linear relationship, and the group average correlation coefficient is not significantly different from zero.

**FIGURE 7:**
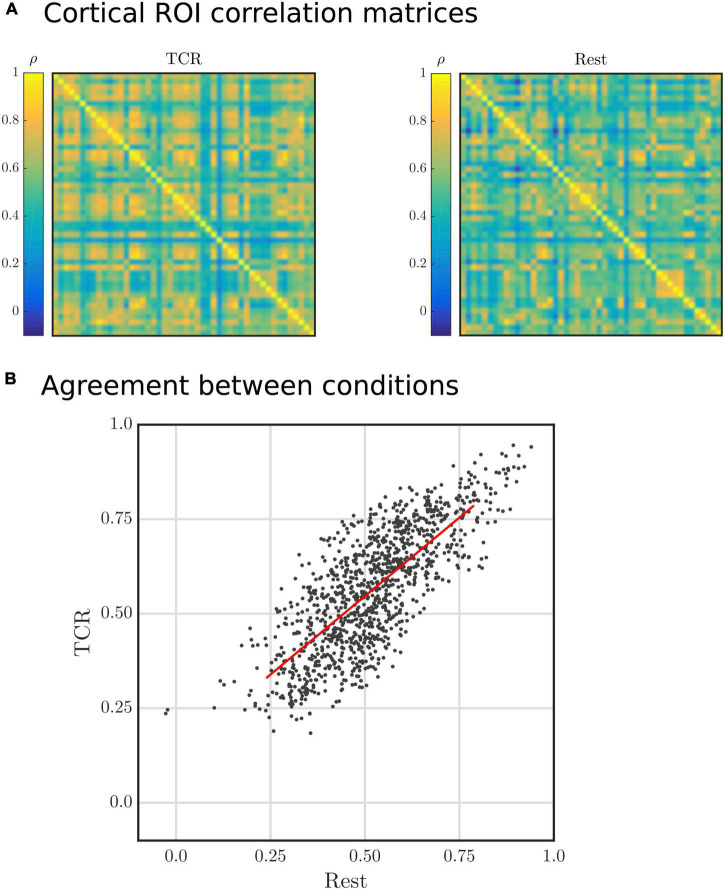
**(A)** Group average Harvard-Oxford cortical ROI correlation matrices for Rest and TCR conditions. The order of the ROIs in the matrices is not particularly important in this physiological context, but can found by referring to the Harvard-Oxford Cortical Atlas. **(B)** Scatter plot of group average Harvard-Oxford cortical ROI correlations between the Rest and TCR condition. Each point represents a correlation between two cortical ROIs for each condition.

Correlation matrices generated from resting-state data, following some parcellation of the brain into presumed neuroanatomically distinct regions, are often used to visualize functional connectivity, which is hypothesized to reflect the macroscopic functional organization of the brain as reflected in the synchronized activity of remote brain regions. The *tcr-fMRI* data are averaged across multiple repeats of the TCR challenge, which removes neutrally driven BOLD fluctuations that are not temporally locked to the TCR challenge. Thus, the *tcr-fMRI* data are expected to contain only variance related to the dCA response, which is fundamentally vascular in nature. Despite this, we still see a large degree of similarity between the rest and TCR correlation matrices, as shown in Figures 7A,B shows a scatter plot of the individual “functional connections,” i.e., correlation between two distinct cortical ROIs, which we can see are strongly positively correlated between the two conditions (Spearman’s rank ρ = 0.76, *p* < 10^–8^).

## Discussion

### General Findings

The aim of this study was to assess the potential of MRI to resolve spatial heterogeneity in dCA, and thus go beyond existing studies that essentially considered only the temporal features. Using a TCR challenge to evoke transient blood pressure changes, we have demonstrated that dCA is associated with distinct spatiotemporal dynamics in a young healthy group of subjects, which we can measure using BOLD fMRI. Consistent with previous fMRI studies we observed a significant drop in the signal following the onset of the TCR (Saeed et al., 2011; Horsfield et al., 2013; Panerai et al., 2016), and as with previous reports we also observed a delayed slow rise in P_*ET*_CO_2_ beginning ∼20 s post-stimulus (Panerai et al., 2015). As this P_*ET*_CO_2_ increase is not associated with any significant change in breathing, it has been suggested that it is due to recirculation of oxygen-depleted and CO_2_-enriched blood trapped in the legs during the occlusion (Panerai et al., 2015). Previous studies have reported increases in HR following TCR that are consistent across subjects (Deegan et al., 2010; Panerai et al., 2015), although without reporting individual subject responses, whereas others have reported a more heterogeneous outcome, albeit in an exercise context (Hildebrandt et al., 1979). As seen in Figure 3C we saw elevated in HR changes in 3 out of 10 subjects, whereas the remainder showed little evidence of an effect. One subject showed a rise and fall in HR prior to TCR, locked instead to the onset of scanning, perhaps as physiological response related to anticipation/anxiety preceding the TCR. Simultaneous continuous blood pressure measurement is a significant challenge in the MR environment, and to our knowledge the Caretaker system is the only commercially available solution at present. We found a significant drop in the Caretaker derived MAP recording following TCR, however it evolves more slowly and lags behind the fMRI signal drop. This is consistent with what we have found previously in endogenous fluctuations (Whittaker et al., 2019b), and likely reflects the fact that the Caretaker blood pressure measure, which is based on analysis of the pulse wave in the thumb, is more sensitive to peripheral vascular changes.

In order to extract spatial dynamics, we employed a voxel-wise fitting procedure using a novel basis set, which allows for a variety of complex response shapes to be characterized in a parsimonious fashion, thus avoiding over-fitting. By adopting this approach, we have shown that the HRF_*TCR*_ exhibits significant regional variability, both with respect to the shape of the curves and its temporal evolution, which determines the relative timing of peaks and troughs. Our results suggest that TCR challenge fMRI is a promising approach for assessing the spatiotemporal features of dCA. The sensitivity of TCD is limited to the major intracranial arteries, which effectively precludes any but the most global of spatial heterogeneities in dCA from being resolved, whereas the high spatial resolution of MRI permits a very fine-grained spatial analysis. Attempts to delineate significant differences in dCA across major vascular territories in healthy individuals have yielded mixed results, but generally suggest any observable differences are small (Rosengarten and Kaps, 2002; Sorond et al., 2005; Reehal et al., 2021). Differences between vascular territories could be more efficiently evaluated with fMRI, as the whole-brain (and thus all vascular territories) can be imaged within in a single scan, in contrast with TCD in which arteries must be individually targeted. However the vascular territories of the major intracranial arteries divide the brain quite neatly, whereas the patterns of clusters we observe appear more complex. Furthermore, the spatial variability in the HRF_*TCR*_ across 48 cortical clusters shares a significant amount of variance with coherence of resting-state fluctuations. This suggests that variability due to the structure of the arterial vasculature originates at finer scale than large intracranial arteries, which supports the notion that fMRI will allow more local vascular determinants of dCA to be assessed.

Although we have previously reported an association between endogenous MAP and fMRI signal fluctuations (Whittaker et al., 2019b), which we suggested likely reflects dCA, the relative merits relying on endogenous fluctuations compared with using a physiological challenge to induce MAP changes is debatable (Simpson and Claassen, 2018; Tzeng and Panerai, 2018). In addition to larger signal changes and increased ecological validity that comes with using a physiological challenge, there is the added benefit that fluctuations not due to dCA can be averaged out. This is more important for fMRI than TCD, as the resting-state fMRI signal is known to contain variance from a wide variety of sources. The more pronounced delineation of the lag structure across layers of WM that we observed in the TCR condition compared with the rest (Figure 5C) is perhaps a testament to this fact. The more purely vascular nature of the tcr-fMRI data in comparison with the rs-fMRI data may mean that the lag structure we observe more closely reflects the passage of blood through the vasculature, as has been observed in response to a CO_2_ stimulus (Bhogal, 2021). Thus, whilst using rs-fMRI data to infer cerebrovascular pathology may be suitable in severe cases such as Stroke (Lv et al., 2013), it may not be appropriate to discern more subtle pathological effects or individual variability within healthy subjects.

### Temporal Dynamics in White Matter

Of particular note is the marked difference in temporal dynamics between the TCR response in GM and WM, which is perhaps to be expected, given the structure of the arterial vasculature. Horsfield et al. (2013) attempted to map some of the spatial variability of the TCR response in fMRI using a two parameter model that accounts for the initial signal drop and subsequent recovery. In agreement with their results, we also found that the magnitude of the signal drop was lower in WM compared with GM. However, they also reported that the rate of recovery parameter in their model implied a quicker return to baseline in WM. Although we can’t compare directly, our results do not support this finding, as we observe what appears to be more slowly evolving dynamics in WM that lag behind the GM response. However, the GM/WM disparity we observe in the TCR response is consistent with a growing literature that reports significant temporal differences in the lag-adjusted correlation between peripheral measures of vascular tone and fMRI signals in the brain (van Houdt et al., 2010; Tong et al., 2013, 2017; Özbay et al., 2018; Chen et al., 2020; Kassinopoulos and Mitsis, 2021). These delayed response patterns in WM, with respect to GM, have been hypothesized to stem from an effect of differential transit times following upstream arterial tone changes, that are related to the specific morphology of WM (Tong et al., 2017; Özbay et al., 2018).

Functional magnetic resonance imaging offers a way to delineate dCA in GM and WM that is simply not possible with TCD, which provides further advantages for its use. Alterations in WM perfusion are associated with numerous pathologies, such as Multiple sclerosis (MS) (De Keyser et al., 2008) and cerebral small vessels disease (CSVD) (Arba et al., 2017). Thus, given that we observe distinctly different temporal dynamics in WM dCA in comparison to GM in our healthy sample, TCR challenge fMRI would appear to present a promising potential diagnostic route.

### Relationship With Resting-State

A topic that has recently attracted interest is the presence of low frequency oscillations in resting-state signals, which are of a presumed systemic physiological origin (Özbay et al., 2018, 2019; Tong et al., 2019; Whittaker et al., 2019b), and for which temporal dynamics show structured spatial variation. The GS in resting-state, which combines multiple sources of variance (Liu et al., 2017), is also sensitive to any sufficiently global flow phenomena in the brain, such as those related to systemic physiological processes. The lag time in lag-adjusted correlations with the GS in resting-state also show structured spatial variance (Amemiya et al., 2016, 2020; Tong et al., 2017, 2019), which is correlated with bolus delivery time in dynamic contrast susceptibility images (Lv et al., 2013; Amemiya et al., 2014; Christen et al., 2015; Tong et al., 2017) and is sensitive to stroke induced haemodynamic changes (Lv et al., 2013; Amemiya et al., 2014). This suggests the time lag information has a predominantly vascular origin, and so reflects the passage of blood through the vascular system (Tong et al., 2019). We found that time lag information was only very weakly correlated between TCR and rest conditions, whereas the pattern of the magnitude of the GS correlation itself was highly correlated.

As the magnitude of the global signal correlation (Figure 5A) has strong GM contrast, it implies that the magnitude of fluctuations primarily reflects local deoxygenated blood volume in both TCR and rest conditions. Conversely, as evidence in Figure 5D, there is no significant correlation between the spatial distribution of the lag times in the TCR and rest conditions. We suggest that this differential lag structure between the two conditions could reflect a difference in the dominant vascular scale (i.e., arterial size) mediating the local hemodynamics, i.e., the TCR response involves the action of larger arteries whose downstream flow response will largely reflect transit time differences related to the morphological difference between GM and WM.

We also observed that functional connectivity matrices were remarkably similar in rest and TCR conditions, despite the fact that we only expect to see neuronal BOLD fluctuations in the rest case. This result is in agreement with previous studies that suggest purely physiological fluctuations show functional network like structure (Bright et al., 2020; Chen et al., 2020). In this study, the averaging together of multiple TCR scans ensures that only task locked neural changes are present, and the majority of signal variance should be related to the predominantly vascular dCA process. It is likely that TCR locked neural activity associated with sensory perception will be present, along with neural responses associated with autonomic regulation evoked by the TCR stimulus. However, as the relevant neural circuits are relatively localized to subcortical regions of the brain, specifically the hypothalamus (Manuel et al., 2020), one would not expect this to account for the large similarity between whole-brain correlation matrices that we observe. Thus, it would appear that a significant proportion of the spatial variance in resting-state correlations and the GS is attributable to something intrinsic to the cerebral vasculature.

### Physiological Interpretation

In this study, we chose to characterize dCA from BOLD fMRI signal changes, which under these experimental conditions we assume are driven purely by CBF changes. However, in reality BOLD signal dynamics reflect the complex interplay of multiple haemodynamic processes (Buxton, 2013) and the state of numerous physiological parameters, such as haematocrit (Levin et al., 2001), basal CBF (Whittaker et al., 2016) and cerebrovascular reactivity (CVR) (Bandettini and Wong, 1997). The complex and non-quantitative nature of the BOLD signal makes interpreting the signal changes non-trivial. Furthermore, a well-known confound with BOLD contrast is its sensitivity to large draining veins downstream from the capillary bed (Turner, 2002), which obfuscates precise localization of flow changes and thus potentially limits its clinical impact. Arterial Spin Labeling (ASL) is an alternative technique that can directly measure and quantify CBF (Fantini et al., 2016), which has been used to study steady-state changes in CBF associated with static CA (Bokkers et al., 2010; van Dalen et al., 2021). ASL can also be used as an fMRI technique to measure dynamic changes in CBF, and it has the distinct advantage of offering a quantitative and direct measure, which in theory is more precisely localized to the capillary bed (Aguirre et al., 2005). However, in practice it suffers from low SNR and lower temporal resolution compared with BOLD, which would likely present a challenge for a dCA application such as in this study. Nevertheless, future research could potentially benefit from using ASL fMRI in order to validate the presumed CBF origin of the BOLD signal changes we have observed.

### Limitations

A main limitation of MRI based dCA research is the availability of appropriate non-invasive continuous blood pressure measurement (Panerai et al., 2016). Although the Caretaker system now exists, its accuracy and reliability compared to the more established Finapres system, which is preferred by the TCD community, remains unknown. In our experience, and as reported by others [MPhil thesis (Hawezi, 2015)], the Caretaker is very sensitive to subject motion and external vibrations, which is further exacerbated by the long connecting tube that connects the pneumatic cuff from the patient in the magnet room with the electronic equipment in the control room. We observed the expected decrease in Caretaker measured MAP, but as in a previous study (Whittaker et al., 2019b) we observed that they lagged behind the TCR evoked fMRI signal changes. For this reason, which automatically precludes methods for quantifying dCA that assume temporal precedence of MAP, we chose not to define any index of autoregulation, but rather have simply reported on the spatiotemporal dynamics and demonstrated the feasibility of voxel-wise analyses. The temporal information present in the BOLD TCR response and the spatial distribution of relative BOLD signal changes will likely be informative in clinical populations, but future research may still focus on how best to integrate blood pressure information to get a more quantitative metric of dCA.

## Conclusion

In this study, we have demonstrated that whole-brain BOLD fMRI has enough sensitivity to measure the voxel-wise HRF_*TCR*_ associated with dCA in a group of healthy young subjects; the group average displaying a distinctive spatiotemporal pattern. In contrast with earlier TCR challenge fMRI studies, the recent wider adoption of both parallel imaging and SMS techniques allow whole-brain coverage with reasonable temporal and spatial resolutions. This makes voxel-wise mapping of dCA and the whole-brain characterization of spatiotemporal dynamics more viable. Furthermore, our finding of a significant structure in spatiotemporal variability in this subject group demonstrate the importance of considering spatial variability in estimates of dCA. Future research should focus on furthering our understanding of the dCA phenomenon in the context of fMRI with an aim to develop clinically useful methods.

## Data Availability Statement

The datasets presented in this study can be found in online repositories. The names of the repository/repositories and accession number(s) can be found below: https://osf.io/exu76/?view_only=9b9227bad3234b0781ce39f4e1d59127.

## Ethics Statement

The studies involving human participants were reviewed and approved by the Cardiff University School of Psychology Ethics Committee. The patients/participants provided their written informed consent to participate in this study.

## Author Contributions

JW and KM conceived of the presented idea. JW, JS, and MV collected the data. JW analyzed the data. All authors provided critical feedback and helped shape the research, analysis, and manuscript.

## Conflict of Interest

The authors declare that the research was conducted in the absence of any commercial or financial relationships that could be construed as a potential conflict of interest.

## Publisher’s Note

All claims expressed in this article are solely those of the authors and do not necessarily represent those of their affiliated organizations, or those of the publisher, the editors and the reviewers. Any product that may be evaluated in this article, or claim that may be made by its manufacturer, is not guaranteed or endorsed by the publisher.
