# Supplementary material for: The Spatiotemporal Dynamics of Cerebral Autoregulation in Functional Magnetic Resonance Imaging
Source: Front Neurosci. 2022 Jul 8;16:795683. doi: 10.3389/fnins.2022.795683 (PMC9304653; doi:10.3389/fnins.2022.795683)
Supplement: Supplementary file 1 [file Data_Sheet_1.pdf]

1. Supplementary material

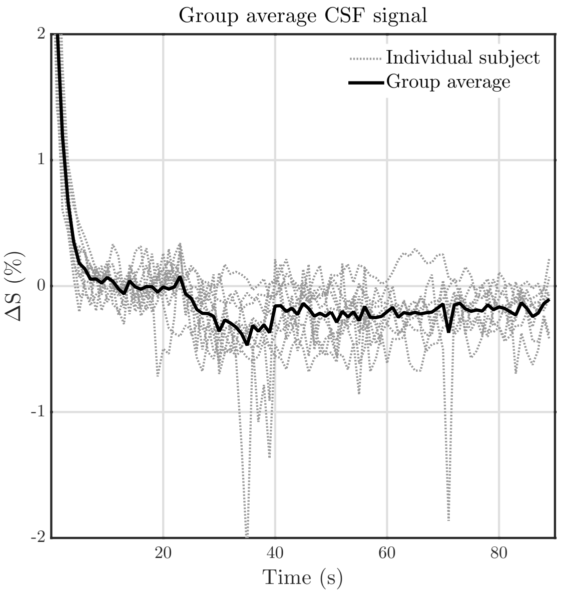

S1: Group average and individual subject lateral ventricle CSF signals. Following TCR onset (20s), there is evidence of a small and sustained signal reduction, but this has only a very small percentage signal change, and there is significant between subject variability. Thus, removal of the CSF signal as part of ANATICOR does not significantly influence the HRF_{TCR} results.

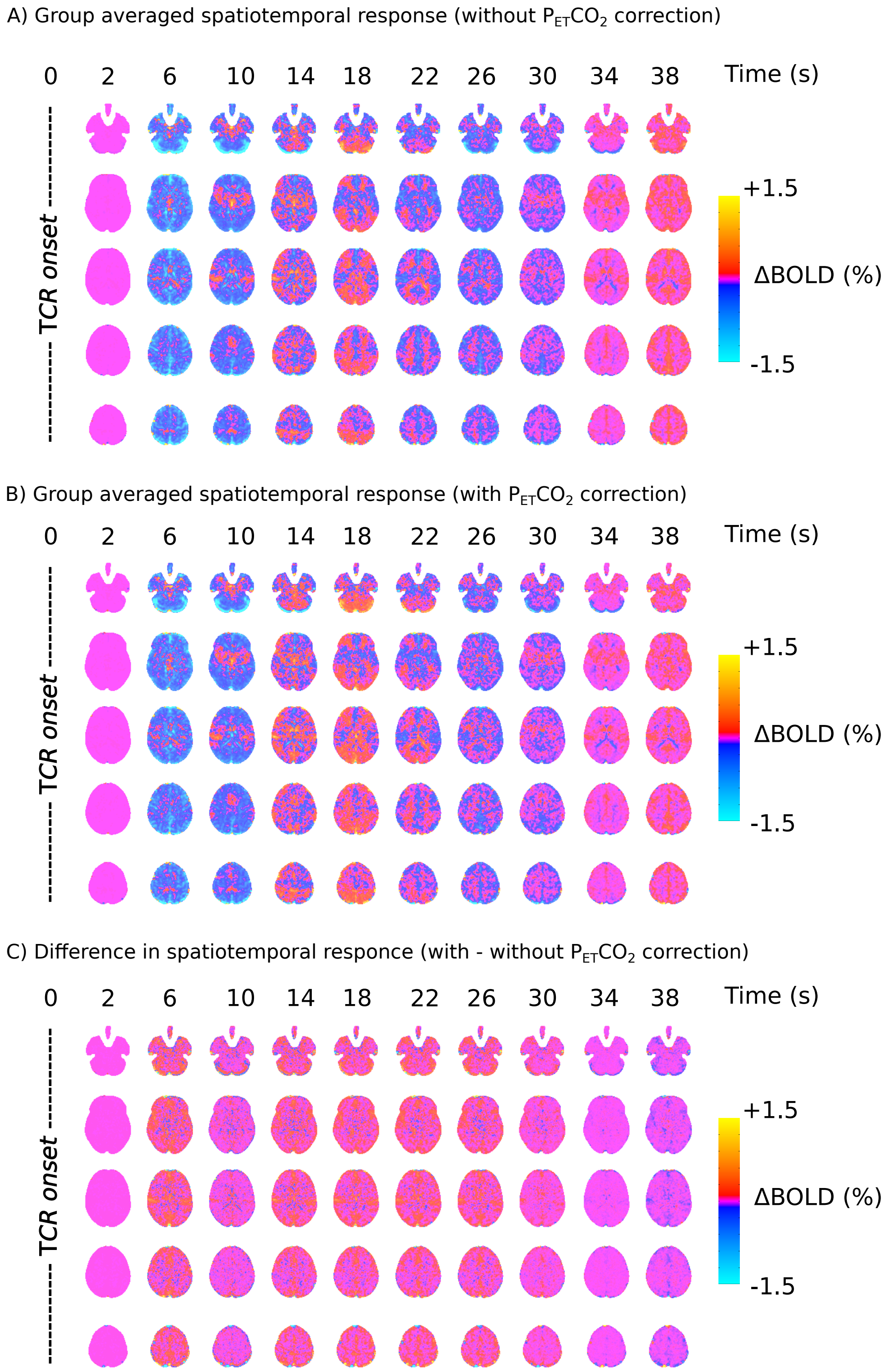

S2: A) Spatiotemporal dynamics of the HRF_{TCR} without including $P_{ET}CO_2$ in model. B) Spatiotemporal dynamics of the HRF_{TCR} with PETCO2 included in model. C) Difference due to including $P_{ET}CO_2$ in model.

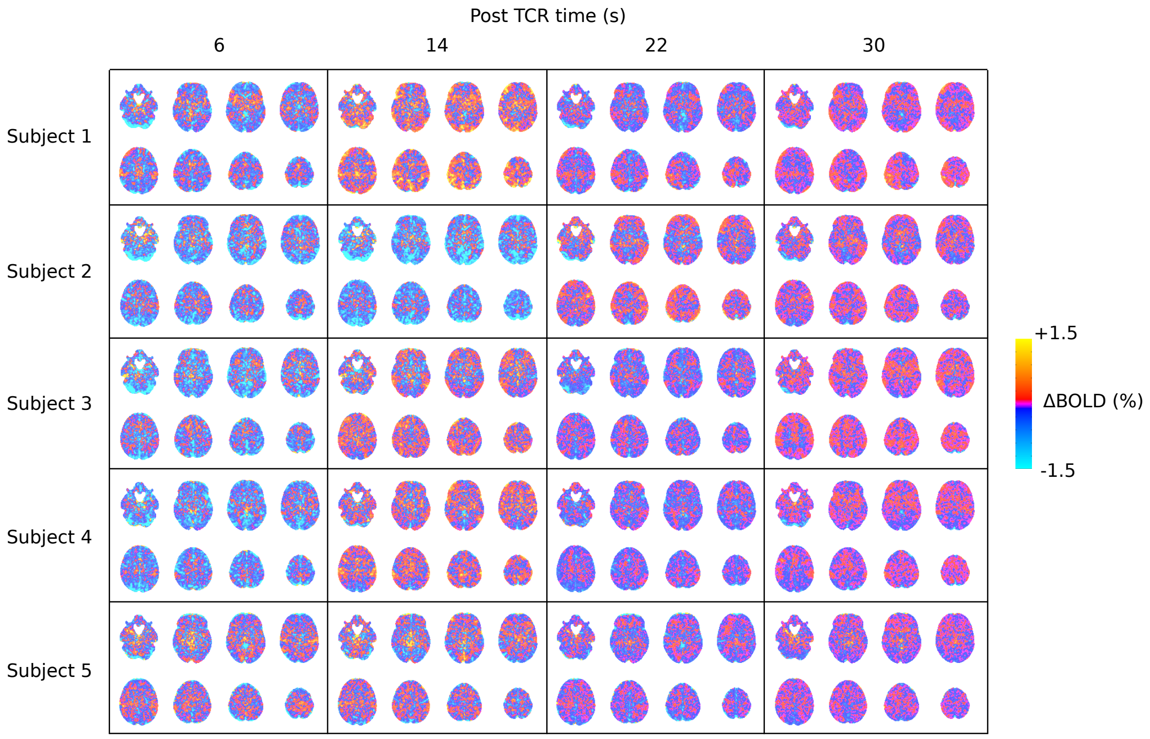

S3: A presentation of the HRF_{TCR} at 4 different time points for the first 5 subjects in order to show the between subject agreement in the spatiotemporal dynamics.

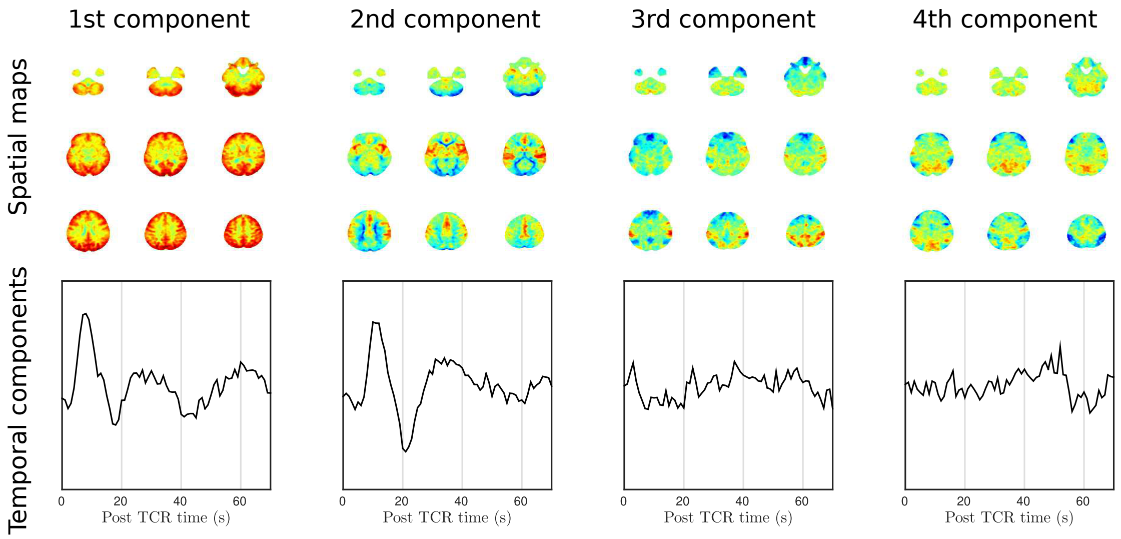

S4: The spatial maps and time series of the top four components following a PCA decomposition of the group averaged data. N.B. The directionality of the temporal components in the PCA output does not necessarily reflect directionality in the original data.
